# Supplementary material for: Modeling glioblastoma heterogeneity as a dynamic network of cell states
Source: Mol Syst Biol. 2021 Sep 16;17(9):e10105. doi: 10.15252/msb.202010105 (PMC8444284; doi:10.15252/msb.202010105)
Supplement: Supplementary file 6 — Source Data for Figure 5 [file MSB-17-e10105-s004.zip › Figure5A_sourcedata/GSEA_3071/nosigdb_stateV.GseaPreranked.1622795159954/GAROFANO_IAVARONE_LIST_OF_NEURAL_PROGENITOR_MARKER_GENES.html]

Details for gene set GAROFANO\_IAVARONE\_LIST\_OF\_NEURAL\_PROGENITOR\_MARKER\_GENES[GSEA]

|  || Dataset | state43071 |
| Phenotype | NoPhenotypeAvailable |
| Upregulated in class | na\_pos |
| GeneSet | GAROFANO\_IAVARONE\_LIST\_OF\_NEURAL\_PROGENITOR\_MARKER\_GENES |
| Enrichment Score (ES) | 0.61896586 |
| Normalized Enrichment Score (NES) | 3.3084023 |
| Nominal p-value | 0.0 |
| FDR q-value | 0.0 |
| FWER p-Value | 0.0 |
Table: GSEA Results Summary

  

Fig 1: Enrichment plot: GAROFANO\_IAVARONE\_LIST\_OF\_NEURAL\_PROGENITOR\_MARKER\_GENES      
 Profile of the Running ES Score & Positions of GeneSet Members on the Rank Ordered List

  

| PROBE | GENE SYMBOL | GENE\_TITLE | RANK IN GENE LIST | RANK METRIC SCORE | RUNNING ES | CORE ENRICHMENT || 1 | ESCO2 |  |  | 3 | 2.193 | 0.0281 | Yes |
| 2 | HMGB2 |  |  | 5 | 2.011 | 0.0542 | Yes |
| 3 | POU3F2 |  |  | 11 | 1.891 | 0.0779 | Yes |
| 4 | DHFR |  |  | 18 | 1.795 | 0.1002 | Yes |
| 5 | RRM2 |  |  | 34 | 1.660 | 0.1190 | Yes |
| 6 | ASCL1 |  |  | 43 | 1.604 | 0.1384 | Yes |
| 7 | PBK |  |  | 54 | 1.536 | 0.1566 | Yes |
| 8 | EZH2 |  |  | 55 | 1.534 | 0.1766 | Yes |
| 9 | INSM1 |  |  | 72 | 1.467 | 0.1927 | Yes |
| 10 | FBXO5 |  |  | 82 | 1.422 | 0.2096 | Yes |
| 11 | HES6 |  |  | 91 | 1.391 | 0.2262 | Yes |
| 12 | DEK |  |  | 94 | 1.384 | 0.2439 | Yes |
| 13 | FABP7 |  |  | 98 | 1.372 | 0.2612 | Yes |
| 14 | RRM1 |  |  | 99 | 1.364 | 0.2791 | Yes |
| 15 | LIG1 |  |  | 145 | 1.284 | 0.2872 | Yes |
| 16 | SOX2 |  |  | 147 | 1.279 | 0.3037 | Yes |
| 17 | MIS18BP1 |  |  | 159 | 1.247 | 0.3179 | Yes |
| 18 | TMPO |  |  | 161 | 1.243 | 0.3339 | Yes |
| 19 | GPD1 |  |  | 177 | 1.219 | 0.3470 | Yes |
| 20 | NASP |  |  | 180 | 1.212 | 0.3624 | Yes |
| 21 | NUF2 |  |  | 192 | 1.189 | 0.3759 | Yes |
| 22 | PRC1 |  |  | 211 | 1.161 | 0.3876 | Yes |
| 23 | UBE2C |  |  | 214 | 1.154 | 0.4023 | Yes |
| 24 | TPX2 |  |  | 248 | 1.122 | 0.4106 | Yes |
| 25 | RAD21 |  |  | 256 | 1.109 | 0.4237 | Yes |
| 26 | CKAP2L |  |  | 285 | 1.078 | 0.4324 | Yes |
| 27 | ARHGAP11A |  |  | 303 | 1.064 | 0.4431 | Yes |
| 28 | PAX6 |  |  | 311 | 1.055 | 0.4555 | Yes |
| 29 | CKAP2 |  |  | 312 | 1.053 | 0.4693 | Yes |
| 30 | DLL3 |  |  | 315 | 1.048 | 0.4826 | Yes |
| 31 | OXCT1 |  |  | 321 | 1.045 | 0.4953 | Yes |
| 32 | PROM1 |  |  | 356 | 1.008 | 0.5019 | Yes |
| 33 | DNAJC9 |  |  | 373 | 0.979 | 0.5116 | Yes |
| 34 | UHRF1 |  |  | 390 | 0.958 | 0.5211 | Yes |
| 35 | SLBP |  |  | 403 | 0.949 | 0.5312 | Yes |
| 36 | KPNA2 |  |  | 435 | 0.919 | 0.5372 | Yes |
| 37 | RACGAP1 |  |  | 443 | 0.912 | 0.5478 | Yes |
| 38 | CLU |  |  | 464 | 0.900 | 0.5557 | Yes |
| 39 | HMGN2 |  |  | 466 | 0.900 | 0.5673 | Yes |
| 40 | SPAG5 |  |  | 481 | 0.884 | 0.5761 | Yes |
| 41 | TCF12 |  |  | 512 | 0.855 | 0.5815 | Yes |
| 42 | TCF19 |  |  | 542 | 0.842 | 0.5870 | Yes |
| 43 | SALL1 |  |  | 545 | 0.836 | 0.5975 | Yes |
| 44 | MYO10 |  |  | 629 | 0.783 | 0.5918 | Yes |
| 45 | TP53I11 |  |  | 653 | 0.772 | 0.5975 | Yes |
| 46 | NDE1 |  |  | 672 | 0.762 | 0.6040 | Yes |
| 47 | NOTCH1 |  |  | 673 | 0.762 | 0.6139 | Yes |
| 48 | ANP32E |  |  | 722 | 0.739 | 0.6144 | Yes |
| 49 | SUZ12 |  |  | 846 | 0.680 | 0.5996 | Yes |
| 50 | SOX9 |  |  | 905 | 0.658 | 0.5971 | Yes |
| 51 | H2AFV |  |  | 933 | 0.647 | 0.6003 | Yes |
| 52 | ARX |  |  | 937 | 0.647 | 0.6082 | Yes |
| 53 | CKS2 |  |  | 966 | 0.634 | 0.6111 | Yes |
| 54 | NBL1 |  |  | 973 | 0.630 | 0.6182 | Yes |
| 55 | TUBA1B |  |  | 1012 | 0.617 | 0.6190 | Yes |
| 56 | DBI |  |  | 1118 | 0.588 | 0.6065 | No |
| 57 | NUCKS1 |  |  | 1178 | 0.570 | 0.6026 | No |
| 58 | ANP32B |  |  | 1187 | 0.566 | 0.6085 | No |
| 59 | HMGN5 |  |  | 1292 | 0.534 | 0.5955 | No |
| 60 | PTBP1 |  |  | 1311 | 0.530 | 0.5989 | No |
| 61 | NES |  |  | 1325 | 0.527 | 0.6033 | No |
| 62 | DLG1 |  |  | 1491 | 0.482 | 0.5779 | No |
| 63 | CKS1B |  |  | 1502 | 0.479 | 0.5823 | No |
| 64 | MSI1 |  |  | 1601 | 0.459 | 0.5694 | No |
| 65 | TUBB4B |  |  | 1659 | 0.444 | 0.5643 | No |
| 66 | KIF2A |  |  | 1857 | 0.409 | 0.5318 | No |
| 67 | ANLN |  |  | 2125 | 0.371 | 0.4854 | No |
| 68 | DLL1 |  |  | 2147 | 0.368 | 0.4861 | No |
| 69 | ZIC1 |  |  | 2150 | 0.368 | 0.4906 | No |
| 70 | PAICS |  |  | 2403 | 0.334 | 0.4465 | No |
| 71 | RPA2 |  |  | 2570 | 0.314 | 0.4188 | No |
| 72 | SFRP1 |  |  | 2591 | 0.312 | 0.4190 | No |
| 73 | AHCY |  |  | 2598 | 0.311 | 0.4219 | No |
| 74 | SAE1 |  |  | 2872 | 0.283 | 0.3732 | No |
| 75 | PMF1 |  |  | 2895 | 0.279 | 0.3726 | No |
| 76 | CALU |  |  | 3001 | 0.269 | 0.3560 | No |
| 77 | RANGAP1 |  |  | 3006 | 0.268 | 0.3587 | No |
| 78 | IRX3 |  |  | 3322 | -0.271 | 0.3017 | No |
| 79 | NUDC |  |  | 3349 | -0.275 | 0.3003 | No |
| 80 | WNT7B |  |  | 3515 | -0.303 | 0.2726 | No |
| 81 | IDI1 |  |  | 3680 | -0.328 | 0.2454 | No |
| 82 | GLI2 |  |  | 3770 | -0.344 | 0.2328 | No |
| 83 | VIM |  |  | 3830 | -0.354 | 0.2261 | No |
| 84 | YBX3 |  |  | 3863 | -0.361 | 0.2246 | No |
| 85 | CLIC4 |  |  | 4075 | -0.404 | 0.1894 | No |
| 86 | NOTCH2 |  |  | 4355 | -0.488 | 0.1422 | No |
| 87 | EFHD2 |  |  | 4542 | -0.559 | 0.1138 | No |
| 88 | GLI1 |  |  | 5101 | -1.156 | 0.0217 | No |
| 89 | FOXA2 |  |  | 5107 | -1.176 | 0.0361 | No |
Table: GSEA details [plain text format]

  

Fig 2: GAROFANO\_IAVARONE\_LIST\_OF\_NEURAL\_PROGENITOR\_MARKER\_GENES: Random ES distribution      
 Gene set null distribution of ES for **GAROFANO\_IAVARONE\_LIST\_OF\_NEURAL\_PROGENITOR\_MARKER\_GENES**
